# Dynamic stem–loop extension by Pol θ and templated insertion during DNA repair

**DOI:** 10.1016/j.jbc.2024.107461

**Published:** 2024-06-12

**Authors:** Denisse Carvajal-Maldonado, Yuzhen Li, Mark Returan, April M. Averill, Sylvie Doublié, Richard D. Wood

**Affiliations:** 1 Department of Epigenetics and Molecular Carcinogenesis, The University of Texas MD Anderson Center, Houston, Texas, USA; 2 Department of Microbiology and Molecular Genetics, University of Vermont, Burlington, Vermont, USA

**Keywords:** DNA repair, DNA synthesis, protein–DNA interaction, RNA synthesis, DNA polymerase

## Abstract

Theta-mediated end joining (TMEJ) is critical for survival of cancer cells when other DNA double-stranded break repair pathways are impaired. Human DNA polymerase theta (Pol θ) can extend ssDNA oligonucleotides, but little is known about preferred substrates and mechanism. We show that Pol θ can extend both ssDNA and RNA substrates by unimolecular stem–loop synthesis initiated by only two 3ʹ terminal base pairs. Given sufficient time, Pol θ uses alternative pairing configurations that greatly expand the repertoire of sequence outcomes. Further primer-template adjustments yield low-fidelity outcomes when the nucleotide pool is imbalanced. Unimolecular stem–loop synthesis competes with bimolecular end joining, even when a longer terminal microhomology for end joining is available. Both reactions are partially suppressed by the ssDNA-binding protein replication protein A. Protein-primer grasp residues that are specific to Pol θ are needed for rapid stem–loop synthesis. The ability to perform stem–loop synthesis from a minimally paired primer is rare among human DNA polymerases, but we show that human DNA polymerases Pol η and Pol λ can catalyze related reactions. Using purified human Pol θ, we reconstituted *in vitro* TMEJ incorporating an insertion arising from a stem–loop extension. These activities may help explain TMEJ repair events that include inverted repeat sequences.

Theta-mediated end joining (TMEJ, mediated by DNA polymerase theta [Pol θ]) is a major pathway for the repair of double-stranded breaks (DSBs) in DNA. TMEJ protects chromosomes against extensive deletions, limits loss of heterozygosity, and is the primary DSB repair pathway for some stages of organism development (1). In somatic cells, TMEJ is particularly important when other repair pathways are impaired (1). Pol θ functions in repair by acting on two ssDNA tails generated at each end of a break (2). At least one of the single-stranded ends acts as a primer to initiate synthesis at a short region of DNA sequence homology (microhomology) on the other single strand.

To understand how Pol θ functions in this and other (3, 4, 5) pathways, it is important to understand DNA synthesis reactions conducted by the enzyme. Human DNA Pol θ has relaxed template requirements compared to other polymerases in the A family. For example, it can readily bypass some template DNA lesions, usually inserting an A opposite an abasic site or thymine glycol in the template (6, 7). The overall error rate is 10- to 100-fold higher than other A-family polymerases, with many of the errors arising from slippage of the template relative to the primer to yield +1 frameshifts in runs of identical bases (8). Further, extension from specific 3′ terminal primer-template mismatches is relatively efficient (9).

Another remarkable feature of Pol θ is its ability to add nucleotides to ssDNA (10). Multiple nucleotides can be added but the basis of this synthesis has not been clear. Some characteristics of the extension of ssDNA suggest that synthesis is largely templated. For example, extension of homopolymeric oligonucleotides greatly favors incorporation of the complementary base, in the presence of Mg^2+^ (11). For extension of mixed-sequence oligonucleotides, products were suggested to arise from a combination of templated extension and slippage (12). In some cases, significant extension can occur with only a single deoxynucleotide (dNTP) provided (10). The factors controlling the initiation and fidelity of ssDNA extension are not known, and not all features are readily explained.

Like other A-family polymerases, Pol θ can use dATP to add an additional A residue to a blunt DNA end, but otherwise shows predictable template requirements in a first round of DNA synthesis (13). In the presence of Mn^2+^, Pol θ extends ssDNA more avidly; it was at first suggested that this arose by terminal transferase activity but subsequent experiments indicate that the extension largely arises by templated incorporation of bases although the exact rules are unknown (12, 14). Given sufficient time, Pol θ can add bases in the presence of Mn^2+^ to yield products that are essentially random (15, 16). This may still arise from more promiscuous templating because of the high frequency of mismatch incorporation promoted by Mn^2+^.

Here, we used purified protein and DNA to determine sequence features and factors regulating stem–loop (SL) extension. We find that human Pol θ has a remarkable ability to rapidly initiate DNA synthesis by self-pairing a few bases of ssDNA or RNA with a variety of substrates and that it does so even when a microhomology is readily available to perform TMEJ. We identify three primer-grasp amino acid residues unique to Pol θ that help coordinate the SL extension reaction. In a survey of other DNA polymerases, we found that the specialized DNA Pol λ and Pol η enzymes can also conduct SL synthesis on some substrates. It is known that Pol θ synthesizes templated insertions during some repair events. We indicate how some of these may arise from SL-mediated DNA synthesis. Pol θ can conduct the SL extension reaction in multiple dynamic configurations, which could improve TMEJ efficiency by diversifying 3′-ends.

## Results

### Pol θ quickly extends ssDNA and RNA

**Figure 1:**
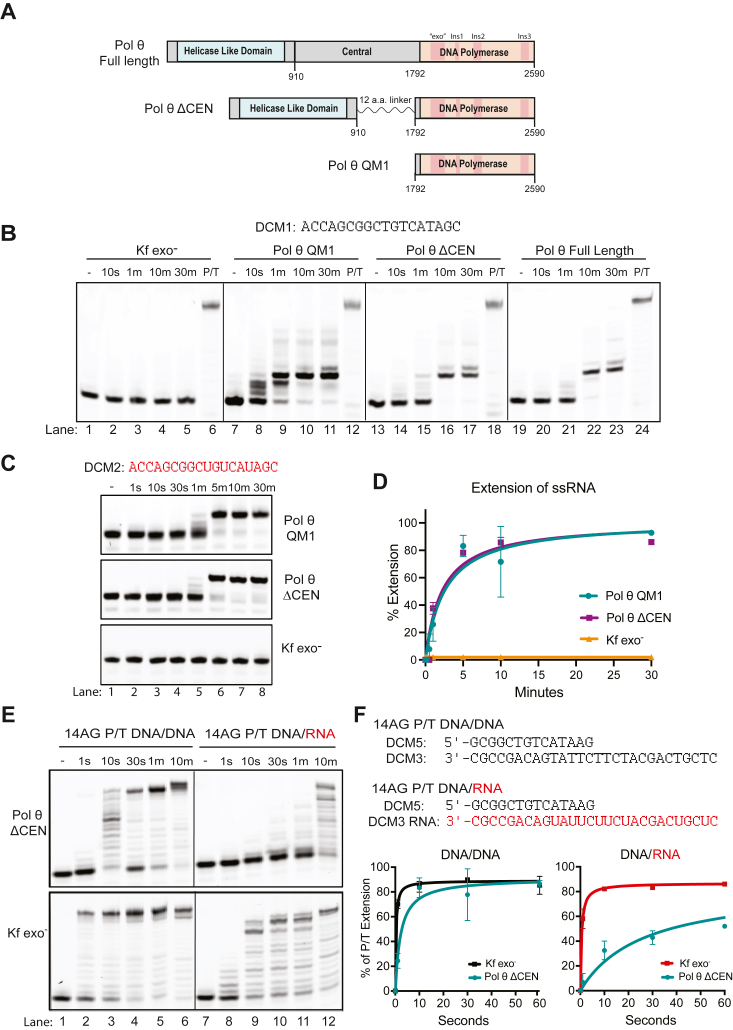
**Pol θ extends ssDNA and ssRNA oligonucleotides to give distinct products.***A*, protein domains and constructs of human Pol θ. *B*, extension of ssDNA oligonucleotide DCM1 using Kf exo^-^, Pol θ QM1, Pol θ ΔCEN, and Pol θ full-length protein. *C*, extension of DCM2 ssRNA by Pol θ QM1 (*top*), Pol θ ΔCEN (*middle*), but not Kf exo^-^ (*bottom*). *D*, densitometric quantification of the bands in Figures 1*C* and S2*D* plotted together as percent extension *versus* time ± SD. *E*, primer/template extension of DNA/DNA (*left*) DCM5/DCM3 and DNA/RNA (*right*) DCM5/DCM3 RNA substrates by Pol θ ΔCEN (*top*) and Kf exo^-^ (*bottom*). *F*, quantification of the bands in Figures 1*E* and S2*C* for each enzyme plotted together as percent extension *versus* time ± SD. Two separate primer/template substrates were used to test primer extension and plotted together. Conditions for all reactions are as follows: 25 nM DNA or RNA, 125 nM enzyme, and 100 μM dNTPs. dNTP, deoxynucleotide; Kf exo^-^, Klenow fragment exo^-^.

A DNA polymerase domain construct of Pol θ (Fig. 1*A*, Pol θ QM1; residues 1792–2590) can add dNTPs to the 3′-ends of ssDNA oligonucleotides (10, 17, 18). We found that this activity is also present in a Pol θ protein construct (Pol θ ΔCEN) that also includes the helicase-like domain (HLD) and a shortened central domain as well as in full-length Pol θ (Fig. 1*A*). Similar specific products form with all the enzyme constructs (Fig. 1*B*), suggesting that ssDNA extension is not coordinated by the HLD or central domains. Oligonucleotides are extended rapidly to a single major product. Because Pol θ QM1 and ΔCEN are more readily purified with high yield, they were used in the remainder of the experiments. Pol θ QM1 can extend oligonucleotides regardless of length and with different 3′ terminal bases (Fig. S1*A*). For all the oligonucleotides that were significantly extended, at least two 3′ bases had the potential to form adjacent base pairs. This is illustrated as unimolecular self-pairing in Fig. S1*B* and verified as such below.

We found that RNA oligonucleotides can also be extended by Pol θ using dNTPs, although more slowly (Figs. 1*C* and S2*D*). Pol θ QM1 and ΔCEN extended two different RNA oligonucleotides with a similar reaction rate, plotted together in Figure 1*D*. By contrast, A homologous bacterial family A DNA polymerase, Klenow fragment exo^-^ (Kf exo^-^) from *Escherichia coli* DNA polymerase I, cannot extend ssDNA or RNA (Figs. 1, *B*–*D* and S2*D*). When ribonucleotides (NTPs) were supplied instead of dNTPs in buffer containing Mg^2+^, human Pol θ QM1 did not extend ssDNA (Fig. S2, *A* and *B*). However, a variant of Pol θ QM1 containing an E2335G mutation in the “steric gate” of Pol θ (15) was able to incorporate NTPs slowly (Fig. S2*B*). Both Kf exo^-^ and Pol θ ΔCEN could also use RNA as a template to extend DNA primers using dNTPs, as previously reported (19, 20). With an RNA template, the rate of DNA synthesis by Pol θ ΔCEN is about 40-fold slower than Kf exo^-^ (Fig. 1, *E* and *F* and S2, *C* and *E*).

When Pol θ processes certain shorter GC-rich oligonucleotides, the products migrate faster than the original oligonucleotides as a compact hairpin form (21, 22, 23) because they do not denature, even on gels containing urea (Figs. S1, *A* and *D* and S2*B*). Previously, the faster migration was interpreted erroneously as evidence for nuclease activity in the Pol θ polymerase domain (18, 24).

### Pol θ extends oligonucleotides by unimolecular synthesis

**Figure 2:**
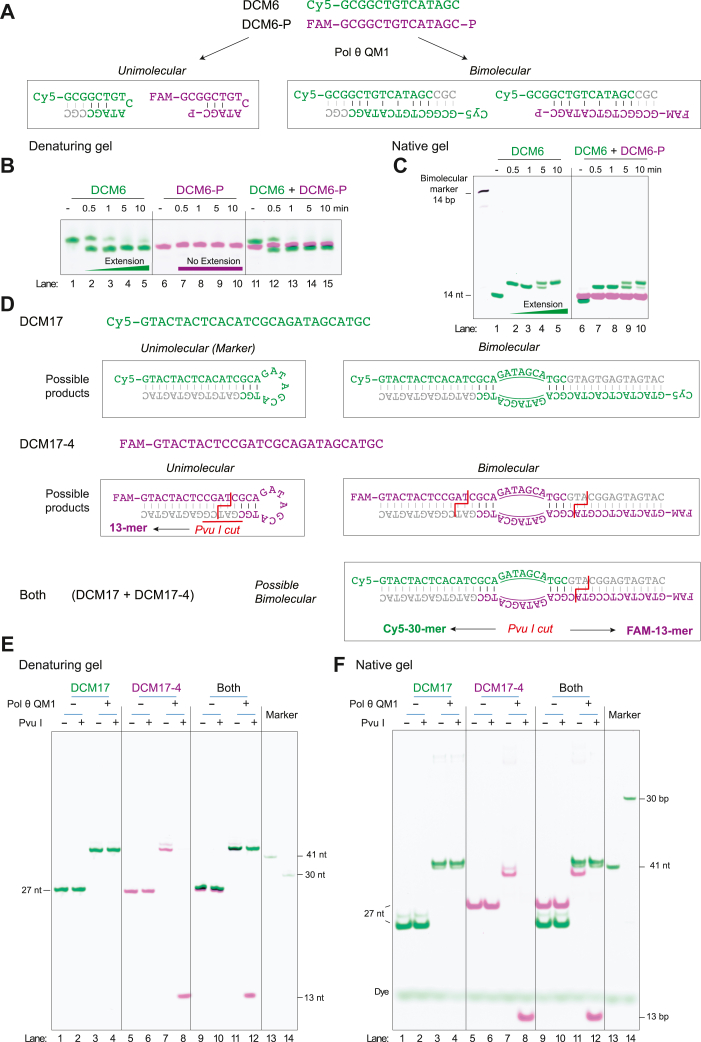
**Extension of short ssDNA oligonucleotides occurs largely by stem–loop synthesis.***A*, two oligonucleotides were analyzed, in reaction mixtures with 125 nM Pol θ QM1, 25 nM DNA, and 100 μM dNTP. DCM6 is 5′-labeled with Cy5. DCM6-P is 5ʹ-FAM-labeled and has a 3ʹ-phosphate group to prevent extension. If DCM6 extends by using another oligonucleotide as template, ∼two-thirds of the products would migrate on a native gel as a 14- to 17-mer double-stranded species containing both fluorophores. *B*, denaturing 20% PAGE gel showing that DCM6 produces an extension product migrating as expected and that DCM6-P does not extend. *C*, nondenaturing 25% PAGE gel showing that extension of DCM6 (*green*) does not arise by pairing with DCM6-P (*purple*), as a paired product would migrate at the 14-bp marker position and contain both fluorophores (*black*). This marker is the annealed product of DCM6 with its complementary FAM-labeled DCM22. *D*, scheme of predicted products derived from DCM17 and DCM17-4 oligonucleotides. Unimolecular and bimolecular products with potential *Pvu*I cleavage sites are shown. If bimolecular pairing were used between DCM17 and DCM17-4, DNA synthesis would yield a 41-bp double strand fragment. *Pvu*I cleavage would yield a ∼30-mer Cy5-labeled fragment and a ∼13-mer FAM-labeled fragment. The *colored text* shows the original oligonucleotide sequence with the predicted synthesis in *gray text*. *E*, DCM17, DCM17-4, or both combined were incubated in reaction mixtures containing 125 nM Pol θ QM1, 25 nM each DNA, and 100 μM dNTPs. On a denaturing 20% polyacrylamide gel, 41-nt products are produced as expected, with the DCM17-4 product susceptible to *Pvu*I cleavage. *F*, on a nondenaturing 25% PAGE gel, the samples migrate similarly. There is no 41-bp species, and no Cy5-labeled 30-mer is produced following *Pvu*I cleavage. This shows that the visible products are synthesized in independent unimolecular reactions. dNTP, deoxynucleotide.

In principle, extension of an ssDNA oligonucleotide might occur by either unimolecular “stem–loop” templating or by hybridization of two copies (Fig. 2*A*). To distinguish between these mechanisms, Cy5-labeled DCM6 was used in conjunction with DCM6-P, a version of DCM6 with a 5′-FAM label and a 3′-phosphate group. The two fluorescent labels distinguish between the products of single-strand extension (purple and green) and bimolecular extension, which appears merged as a black band in a nondenaturing gel. Following incubation with Pol θ QM1, DCM6 extended to give the expected product on a denaturing gel, while DCM6-P, lacking a 3ʹ OH, did not extend (Fig. 2*B*, lanes 1–10). A reaction mixture containing both oligonucleotides gave the same two independent products (Fig. 2*B*, lanes 11–15). To find out whether these products were generated in a bimolecular or a unimolecular reaction, the same reaction mixtures were separated on a nondenaturing gel (Fig. 2*C*). If extension is accomplished by biomolecular annealing as diagrammed in Figure 2*A* (right), most extended products would migrate near the biomolecular 14-bp marker, in a band containing both fluorophores (black in this color scheme). Figure 2*C* shows that no corresponding paired product is present. This shows that DCM6 was extended independently in a unimolecular reaction.

A second approach was designed to distinguish between unimolecular and bimolecular reactions. This scheme utilized 27-mers: Cy5-labeled DCM17 and FAM-labeled DCM17-4 (Fig. 2*D*). After incubation with Pol θ QM1, analysis on a denaturing gel showed that both oligonucleotides were extended (Fig. 2*E*). Cleavage with *Pvu*I restriction enzyme generated a FAM-labeled 13-mer arising from a *Pvu*I recognition sequence in DCM17-4 (Fig. 2*E*, lanes 8 and 12). If extension was accomplished by biomolecular annealing as diagrammed in Figure 2*D* (right), then fully extended products would migrate on a nondenaturing gel as a 41-bp double-stranded fragment containing both fluorophores. Cleavage of this product would produce a predicted Cy5-labeled 30-mer. However, no 41-bp product is formed and no 30-bp Cy5-labeled product is formed by *Pvu*I cleavage of products from reaction mixtures containing the two oligonucleotides (Fig. 2*F*). Instead, products are formed by unimolecular synthesis because their major products migrate near the unimolecular 41-nt marker (Fig. 2, *E* and *F*), and a 13-bp product is formed after *Pvu*I cleavage of reaction mixtures containing DCM17-4 (Fig. 2*F*, lanes 8 and 12). This shows that unimolecular synthesis is the major mode of extension for these short oligonucleotides.

### Unimolecular SL extension by Pol θ is more efficient with two terminal base pairs

**Figure 3:**
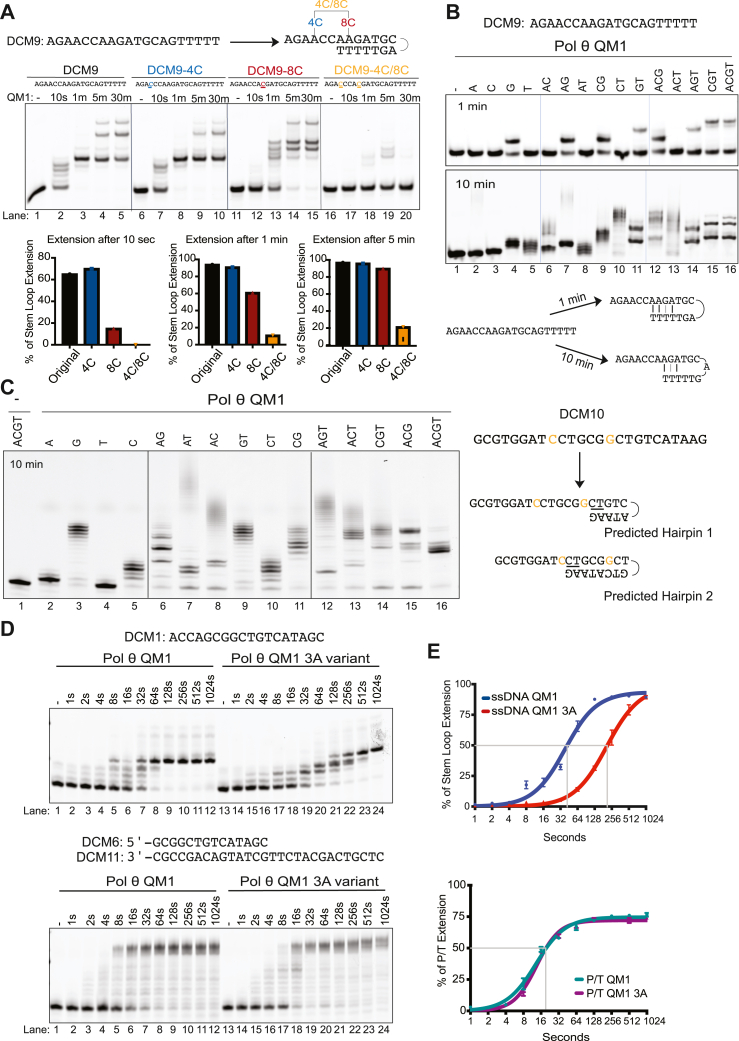
**Factors governing stem–loop extension.***A*, stem–loop extension is suppressed by altering preferred base-pairing sites (*top*). Quantification of the percent of stem–loop extension for each substrate at different time points (*bottom*). Conditions: 25 nM DNA, 125 nM Pol θ QM1, and 100 μM dNTPs. *B*, Pol θ QM1 stem**–**loop extension of DCM9 using different combinations of dNTPs in a 1-min reaction and a longer 10-min reaction. Schematic of hairpins used by Pol θ QM1 in the short and longer reactions. *C*, Pol θ QM1 stem**–**loop extension of DCM10 using different combinations of dNTPs in a 10-min reaction (*left*). Conditions: 25 nM DNA, 125 nM Pol θ QM1 (except lane 1, no enzyme), and 100 μM each of the indicated dNTPs. Schematic of the predicted hairpins used by Pol θ QM1 to extend in the reactions containing just dGTP or dCTP (*right*). *D*, stem–loop extension of DCM1 ssDNA (*top*) and a primer template substrate (*bottom*) using Pol θ QM1 and Pol θ QM1 3A variant. Conditions: 25 nM DNA, 50 nM Pol θ QM1 or 3A variant, and 100 μM dNTPs. *E*, densitometry quantification of the gel bands in (*D*) for each enzyme plotted as percent stem–loop extension *versus* time in seconds for the reactions using ssDNA (*top*) and the primer-template (*bottom*). The experiment was run three times, and the combined data were plotted as described in Experimental procedures. With primer-template substrate K_half_ was the same for the two enzymes, but with stem–loop substrate K_half_ = 38 for Pol θ QM1 and 209 for Pol θ QM1 3A, a time lag of 171 s (∼3 min). dNTP, deoxynucleotide.

Given the mode of extension described above, we refer to templated unimolecular extension as “SL synthesis.” Experiments using a set of oligonucleotides with different sequences suggest that such synthesis can be initiated by two or three base pairs at the 3′ end (Fig. S1*B*). SL extension is more efficient with two 3′ base pairs than one. The 3ʹ-TT sequence of DCM9 can potentially pair at either of two internal AA sequences (Fig. 3*A*). We designed three oligonucleotides based on DCM9 that would reduce pairing at each site from two to only one terminal base pair (Fig. 3*A*). Disrupting one potential pairing site with an A to C mutation (4C) had no major effect (Fig. 3*A* lanes 1–10). Disrupting the other potential pairing site (8C) with an A to C mutation greatly slowed synthesis (Fig. 3*A* lanes 11–15), indicating that 8C is in the primary pairing site. Abolishing this preferred pairing site forces Pol θ QM1 to extend slowly using alternative pairing. When both pairing sites had only one base pair available, SL extension was almost completely blocked (Fig. 3*A*, lanes 16–20).

### Pol θ SL extension operates in a fast, precise mode and a slower mode with slippage

When Pol θ′s ability to extend ssDNA was first reported, several features were puzzling and required further investigation. Notably, Pol θ can extend some short mixed-sequence oligonucleotides even if supplied with a single dNTP (10). This suggested that Pol θ might catalyze nontemplated addition to DNA in some way. On the other hand, if single dNTPs were supplied to Pol θ for synthesis on a homopolymeric DNA substrate, only the complementary dNTP was incorporated. For example, only dATP was incorporated opposite oligo (dT)_20_, and only dGTP opposite oligo (dC)_20_ (10). This indicated that addition of nucleotides was largely templated.

In considering these data, we thought it possible that incorporation by Pol θ initially follows templating rules and that lower fidelity or nontemplated synthesis occurs during longer incubation times. To probe this issue, DCM9 ssDNA was incubated with Pol θ QM1 and specific individual dNTPs for 1 min (fast) or 10 min (slow) (Fig. 3*B*). When a single dNTP was provided, only dGTP allowed Pol θ to extend the ssDNA after 1 min. Two G nucleotides were incorporated before stalling (lane 4), consistent with the SL pairing indicated below the gel image. The reaction that contained both dGTP and dTTP extended further, adding GGTT (lane 11). Finally, the reactions containing dGTP, dTTP, and dCTP extended to the end of the template (Fig. 3*B*, lanes 15 and 16). Thus, during a relatively short 1 min incubation time, extension by Pol θ apparently took place by following normal base-pairing rules, self-templating on a SL structure.

In the longer 10-min reactions, many additional products were formed. Some combinations of two dNTPs could give rise to products longer than those with all four dNTPs present. Such products may be accounted for by two previously noted abilities of Pol θ. First, Pol θ can extend from some terminal mismatches (9, 10). Second, Pol θ frequently incorporates additional nucleotides in runs of identical bases (8). Such a reaction takes place by slippage of the primer relative to the template. Note, for example, the reactions with only dTTP present (Fig. 3*B*, lane 5). After 1 min, no T has been incorporated, consistent with the absence of an A at the next templating position for this SL pairing. After 10 min, limited T incorporation occurs. This could take place if Pol θ QM1 uses an alternative less stable pairing site as illustrated at the bottom of Figure 3*B*.

We propose that the smeared products in the gel arise from template-primer slippage and some mismatch extension. Given enough time, Pol θ can reorient the DNA, sometimes in multiple shifts of the 3′-end to give repeated incorporation of the same base. This lower-fidelity mode of incorporation requires more time (occurring here in the 10-min reactions) and takes place especially when all four dNTPs are not available at the same time (Fig. 3*B*). An illustration with another oligonucleotide is shown in Fig. S3*A*. Here, DCM1 may pair at two alternative internal sites. When the dNTP pools are imbalanced, several prominent products of extension are apparent after 1 min, but after 10 min there are multiple products as shown by smeared patterns of extension and generation of longer products. When all four dNTPs are present in equimolar amounts, the SL structure fills to the end and does not undergo further random extension (Fig. S3*A*, lanes 15 and 16).

Because Pol θ sometimes uses alternative pairing sites and can mediate slippage and incorporate mismatches when dNTP choice is limited, many different outcomes arise in extended-time reactions with mixed sequence oligonucleotides. For example, the oligonucleotide DCM10 contains two predicted pairing sites for SL extension, where the next templated base is either a G or a C (Fig. 3*C*). In the presence of dGTP only, Pol θ QM1 can add ten or more nucleotides (lane 3), and when only dCTP is available can add three to five nucleotides (lane 5). This may be accounted for if Pol θ repositions the primer by slippage back on the template after a first incorporation, resulting in repeated insertion of the same base. After 10 min, different combinations of dNTPs lead to incorporation of multiple bases and products that appear as smeared bands (Fig. 3*C*, lanes 7, 8, 12, and 13). Again, when all 4 nt are present, Pol θ QM1 produces a tight reproducible product that is about 10 bases longer than the starting oligonucleotide (lane 16). In this case, Pol θ rapidly extends from preferred hairpin sites all the way to the end of the oligonucleotide without extensive slippage of the 3′-end in the active site.

### Primer grasp residues in the Pol θ active site help coordinate SL extension

In comparison to other A-family DNA polymerases, Pol θ must contain structural elements that promote the ability to manipulate the oligonucleotide into a SL and use it for synthesis. Five basic residues in Pol θ, termed primer-grasp residues, contact backbone phosphates near the 3′-primer end (25) and could help facilitate the folding of ssDNA in the enzyme. Three of those residues are unique to Pol θ orthologs and are not found in other A-family DNA polymerases, including its closest mammalian homolog, Pol ν. We probed whether these three residues were important for SL extension by changing all three to alanine (R2254A, R2202A, K2181A) in Pol θ QM1. We found that the Pol θ QM1 3A primer-grasp variant shows a significant time lag in initiating synthesis of ssDNA (Fig. 3*D*). The Pol θ QM1 3A variant lagged ∼3 min behind in ssDNA extension reactions, although its ability to extend a primer template is not compromised (Fig. 3*E*). These data indicate that basic primer-grasp residues promote the ability to manipulate the oligonucleotide into a SL and use it for synthesis. Once the oligonucleotide is placed in the proper conformation, DNA synthesis can proceed because the catalytic center of the enzyme is unaffected in the 3A variant.

We tested whether the strand slippage activity of Pol θ is facilitated by the primer grasp residues. The Pol θ QM1 3A variant was able to slowly incorporate a single templated G base in DCM10 oligonucleotide, but its ability to add a run of G’s was greatly reduced compared to WT protein, even after 30 min (Fig. S3*B*). This suggests that the 3A variant is less able to mediate primer-template slippage.

### SL extension is rare among human DNA repair polymerases

**Figure 4:**
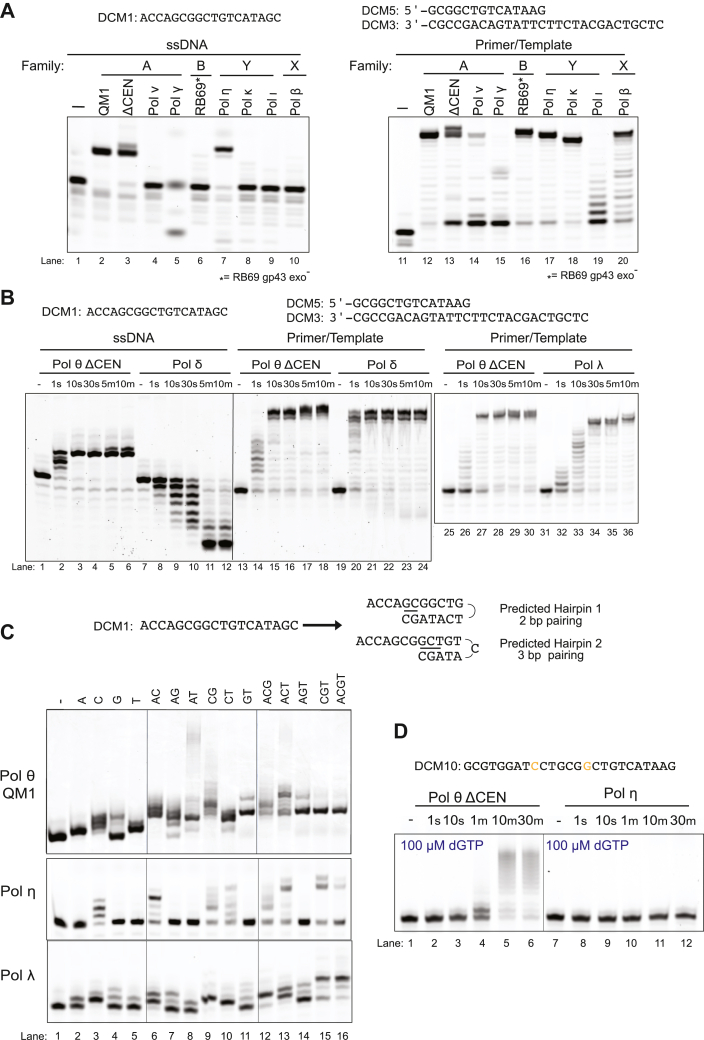
**Stem–loop extension activity on ssDNA is confined to specific specialized DNA polymerases**. *A*, stem–loop extension of DCM1 (*left*) and primer-template (*right*) using several DNA polymerases, Pol θ QM1, Pol θ ΔCEN, Pol ν, Pol γ, RB69 (exo^-^), Pol η, Pol κ, Pol ι, and Pol β. Conditions: 25 nM DNA, 125 nM enzyme, and 100 μM dNTPs. Reactions were incubated for 10 min at 37 °C. *B*, stem–loop extension of DCM1 (*left*) and primer-template (*right*) using Pol θ ΔCEN, Pol δ, and Pol λ at various time points. *C*, stem–loop extension of DCM1 using Pol θ QM1, Pol η, and Pol λ in the presence of different combinations of dNTPs in a long 10-min reaction. Conditions: 25 nM DNA, 125 nM Pol δ/Pol θ QM1/Pol λ, and 100 μM dNTPs. *D*, stem–loop extension slippage activity by Pol θ ΔCEN (*left*) and Pol η (*right*). Conditions: 25 nM DNA, 125 nM enzyme, and 100 μM dGTP. dNTP, deoxynucleotide; RPA, replication protein A; TMEJ, theta-mediated end joining.

To determine whether other specialized human DNA polymerases can initiate SL synthesis, we examined extension of DCM1 with other polymerases. Enzymes were tested from the A-family (Pol ν, Pol γ, and *E. coli* Kf exo^-^), B-family (RB69 gp43 exo^-^ and Pol δ), Y-family (Pol η, Pol κ, and Pol ι), and X-family (Pol β and Pol λ). All were able to extend a primer/template substrate at least partially (Figs. 4, *A* and *B* and S4). Pol θ QM1 was the only tested A-family polymerase capable of SL extension (Figs. 4*A* and S4). Pol ν, like *E. coli* Kf exo^-^, did not alter the ssDNA oligonucleotide. Pol γ has 3′-5′ exonuclease activity (26, 27) and degrades but does not extend ssDNA. Pol δ (B-family) also rapidly degraded ssDNA using its 3′-5′ exonuclease activity, rather than extending it (Fig. 4*B*). The Pol δ ortholog RB69 gp43 exo^-^ also cannot extend ssDNA.

Y-family polymerases Pol κ, Pol ι, and the X-family polymerase β also did not act on an ssDNA oligonucleotide (Fig. 4*A*, lanes 8, 9, and 10), but both Pol η (Y-family) and Pol λ (X-family) were able to extend it (Fig. 4, *A*, lane 7 and *C*). The major products formed by these DNA polymerases may be different in sequence as their migration was not identical to that of the products formed by Pol θ.

Compared to Pol θ QM1, Pol η and Pol λ give rise to a less diverse population of products when extending ssDNA (Figs. 4*C* and S4*B*). In reaction mixtures containing DCM1, Pol η was able to extend with dCTP only, incorporating 3-Cs in a pattern suggesting that little strand slippage occurs (Fig. 4*C*). Unlike Pol θ, Pol η was completely unable to extend the DCM10 oligo when only dGTP is present in the reaction (Fig. 4*D*), suggesting a limited capacity for cycles of strand slippage and incorporation. Pol λ also had a limited capacity for single-strand extension in the presence of single dNTPs, usually adding only one or two nucleotides (Figs. 4*C* and S4*B*).

### SL extension competes with TMEJ *in vitro*

**Figure 5:**
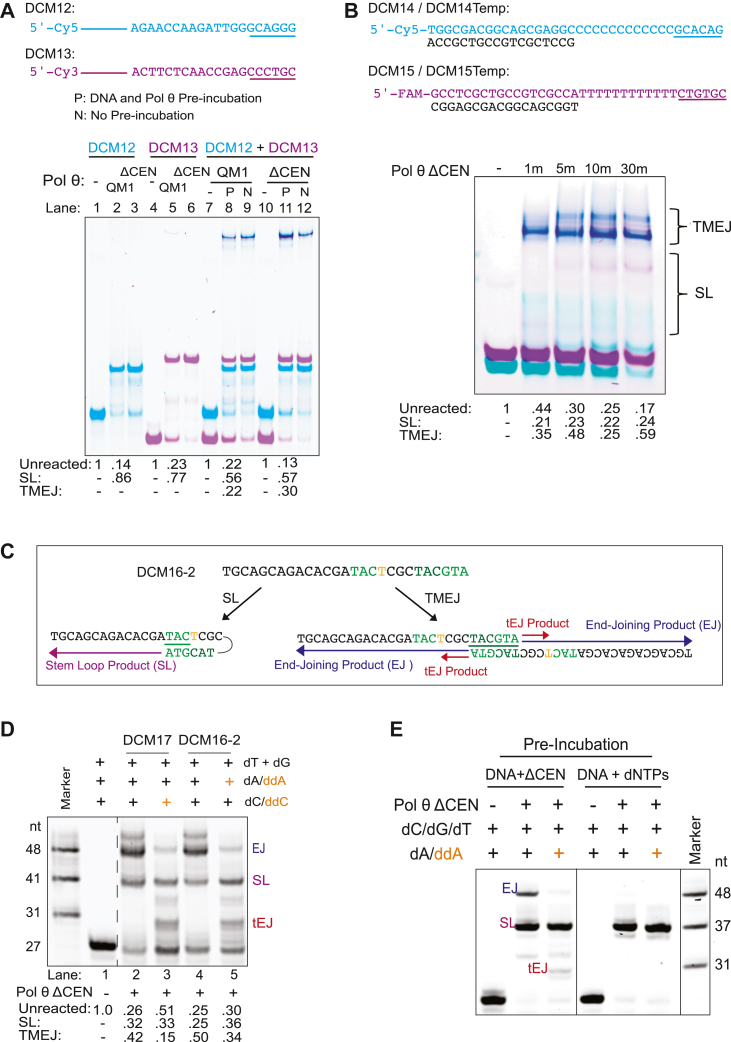
**Competition between TMEJ and stem–loop extension.***A*, TMEJ activity assay using Cy5-labeled DCM12 and Cy3-labeled DCM13. The two 90-mers have a 6-nt 3′ microhomology. DNA was either preincubated (lanes P) with each enzyme for 10 min at 37 °C prior to addition of dNTPs or not preincubated (lanes N). Stem–loop extension using Pol θ QM1 (lanes 2 and 5) or Pol θ ΔCEN (lanes 3 and 6) with each individual 90-mer. TMEJ activity using Pol θ QM1 lanes (lanes 7–9) and Pol θ ΔCEN (lanes 10–12). Conditions: 50 nM of each oligo, 100 nM Pol θ QM1 or Pol θ ΔCEN, and 50 μM dNTPs. Gel was scanned for each fluorescent emission and the images were then merged and artificially colored using ImageJ software (https://imagej.net/software/imagej/). *Cyan* reflects Cy5 gel scan, *purple* reflects the Cy3 gel scan, and *dark blue* is the result of superimposed oligonucleotides and acts a read out for TMEJ. Densitometry quantification of fraction of products formed by Pol θ QM1 and Pol θ ΔCEN is labeled at the *bottom*. *B*, TMEJ activity of DCM14/DCM14Temp and DCM15/DCM15Temp using Pol θ ΔCEN. Conditions: 50 nM of each oligo, 100 nM Pol θ ΔCEN, and 50 μM dNTPs. DCM14 and DCM15 are 36-mer oligonucleotides with a 6-nt microhomology at the 3′-end. DCM14 has 12 consecutive C nucleotides and DCM15 has 12 consecutive T nucleotides prior to the 6-nt microhomology, which limits stable stem–loop extension pairing sites. *Colors* as in panel *A* except FAM has the same color as Cy3. Densitometry quantification of fraction of products formed Pol θ ΔCEN is labeled at the *bottom*. *C*, schematic of Cy5 5′-labeled 27-mer (DCM16-2) for TMEJ *versus* stem–loop extension competition assay. Potential products formed by Pol θ are labeled as a TMEJ product (EJ) in *dark blue*, a stem–loop extension product (SL) in *purple*, and a terminated TMEJ product (tEJ) in *red*. *D*, denaturing gel showing products of TMEJ and stem–loop extension in a mixture of T-27-mer (DCM16-2) and G-27-mer (DCM17). The *dotted line* following lane 2 indicates where four lanes were spliced from the gel. Conditions: 25 nM of each oligonucleotide, 200 nM Pol θ ΔCEN, and 100 μM dNTPs/ddNTPs. Densitometry quantification of the fraction of products formed by Pol θ ΔCEN is labeled at the *bottom*. *E*, TMEJ *versus* SL competition following preincubation for 15 min at 37 °C with Pol θ ΔCEN. Preincubation of DCM16-2 with Pol θ ΔCEN, before addition of dNTPs (*left three lanes*) enhanced TMEJ activity. Preincubation of DNA with dNTPs before addition of Pol θ ΔCEN enhanced stem–loop extension at the expense of TMEJ. dNTP, deoxynucleotide; TMEJ, theta-mediated end joining.

Pol θ operates in DSB repair when a microhomology is available for end joining, and it is important to understand the conditions under which Pol θ performs SL extension, which is expected to compete with end joining. We designed two 90-mer oligonucleotides with a 6-nt terminal microhomology, one oligonucleotide labeled with 5′-Cy5 (cyan) and the other with 5′-Cy3 (purple). When incubated with Pol θ individually, each oligonucleotide formed a major SL product (Fig. 5*A*, lanes 1–6). In reactions including both oligonucleotides, the extended TMEJ products appear as merged color dark blue (lanes 8–9 for Pol θ QM1 and 11–12 for Pol θ ΔCEN). Self-annealing of oligonucleotides competes with TMEJ in such reactions, as shown previously (14, 28). Even with a 6-nt GC-rich terminal microhomology, only about one-fourth of the DNA was used in TMEJ (∼30% of ΔCEN and ∼22% of QM1), while most of the rest of the substrate was consumed by SL extension (see bottom of Fig. 5*A*). Incubating Pol θ ΔCEN with the oligonucleotides before adding dNTPs to initiate the reaction enhanced TMEJ yield (Fig. 5*A*, lane 11 *versus* 12).

To limit the formation of stable hairpins and favor the formation of TMEJ products, we designed 36-nt substrates with a 6-nt terminal microhomology preceded by a run of either 12-Ts or 12-Cs (Fig. 5*B*), flanking a double-stranded region. This substantially increased the percentage of TMEJ products (∼54–59%) after 30 min (Fig. 5*B*). About 20% of the oligonucleotides still undergo unimolecular extension, though the products formed are not discrete and appear as smeared bands (Fig. 5*B*).

To measure competition between SL extension and TMEJ in a different way, a strategy was designed to visualize both processes on a denaturing gel using a single oligonucleotide (Fig. 5*C*). The 27-mer DCM16-2 ssDNA has a palindromic 3′-end that can form a 6-nt terminal microhomology with a second copy of the oligonucleotide to give a TMEJ product (EJ) of 48 nt. The oligo also contains an internal 3-bp site to produce a SL product of 41 nt. To confirm that the 48-nt product arises from TMEJ, the oligonucleotide was designed so that a single ddATP will terminate extension *via* TMEJ but not SL synthesis (Fig. 5*D*). Pol θ ΔCEN extends the 27-mer by both mechanisms, with TMEJ accounting for ∼50% of the products (Fig. 5*D*). Addition of ddATP to reaction mixtures with DCM16-2 produced the predicted 31-nt terminated TMEJ (tEJ) product and diminished the 48-nt TMEJ band, without affecting SL extension (Fig. 5*D*). Addition of ddCTP to reaction mixtures with DCM17 had equivalent consequences. Preincubation of Pol θ ΔCEN with DNA before addition of dNTPs promotes TMEJ (Fig. 5*E*). The results emphasize that with purified DNA, a TMEJ reaction can compete with a unimolecular SL reaction. Recent single-molecule FRET analysis confirms that these reactions are in competition (29).

### RPA suppresses extension

**Figure 6:**
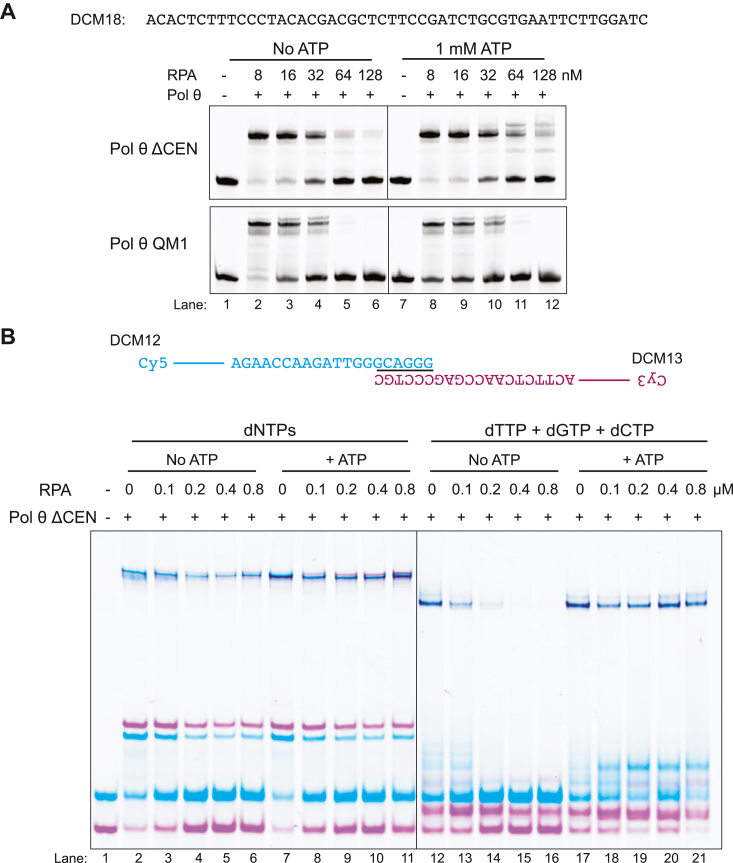
**RPA inhibits stem–loop extension and TMEJ.***A*, stem–loop extension of 50-mer ssDNA (DCM18, 6.4 nM) using Pol θ ΔCEN and Pol θ QM1 in the presence of increasing RPA (8, 16, 32, 64, and 128 nM). Conditions: 6.4 nM DNA, 64 nM Pol θ QM1, or Pol θ ΔCEN and 4 μM dNTPs. DNA and RPA were preincubated for 15 min at 37 °C, before addition of Pol θ ΔCEN ± 1 mM ATP and dNTPs (4 μM). *B*, TMEJ activity assay of two 90-mer paired oligonucleotides (DCM12 and DCM13, 25 nM each) coated with RPA. DNA (25 nM) and increasing concentrations of RPA (0, 0.1, 0.2, 0.4, and 0.8 μM) were preincubated for 15 min at 37 °C, before addition of 200 nM Pol θ ΔCEN ± 5 mM ATP. Reactions were prepared with 100 μM dNTPs (*left*) or 100 μM dTTP/dGTP/dCTP (*right*). dNTP, deoxynucleotide; RPA, replication protein A; TMEJ, theta-mediated end joining.

In cells, the abundant replication protein A (RPA) is generally the first protein to coat ssDNA during DNA transactions (30). Using two 50-mer oligonucleotides, we tested whether RPA could block SL extension. Since an RPA heterotrimer has a footprint on DNA of about 25 to 30 nt (31), a 50-mer oligonucleotide can be covered by only one complete RPA molecule. RPA has a stronger affinity for the 5′-end of DNA (32, 33), so the 3′-end may be accessible for TMEJ. We observed that SL extension by Pol θ decreased with increasing RPA concentration, with high RPA: DNA ratios greatly inhibiting SL extension (Fig. 6*A*). Pol θ is capable of displacing RPA from ssDNA in the presence of ATP using the ATPase activity present in the HLD (34, 35). When Pol θ ΔCEN was provided with 1 mM ATP, more RPA was required to suppress SL extension (Fig. 6*A*).

We tested whether RPA could suppress SL extension of longer oligonucleotides and promote end joining. We used two 90-mers that are prone to SL extension (Fig. 6*B*). Three RPA molecules are required to fully coat a 90-mer. Increased RPA decreased SL extension and TMEJ products (lanes 2–6). When all four dNTPs were present in the reactions, a modest block to TMEJ was observed. Addition of 1 mM ATP restored TMEJ in the presence of RPA (lanes 7–11). The Pol θ ATPase activity can also utilize dATP (36). Thus, a stronger RPA-mediated suppression of TMEJ was observed when dATP was omitted from reaction mixtures (lanes 12–16). Addition of 1 mM ATP fully restored TMEJ, as Pol θ ΔCEN uses the ATP to displace RPA (Fig. 6*B*, right). SL extension is also partially recovered.

### SL extension can diversify 3′-ends and create new microhomologies

**Figure 7:**
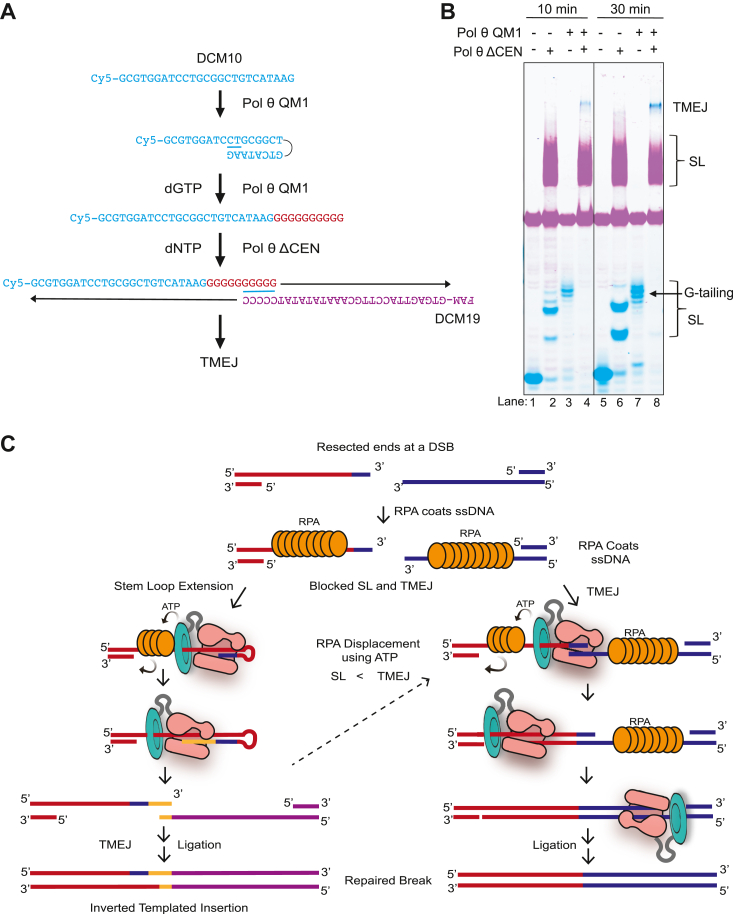
**Reconstitution of a TMEJ product incorporating an inverted repeat.** Schematic (*A*) and gel scan (*B*) of Cy5-labeled DCM10 and FAM-labeled DCM19 oligonucleotides. The two oligonucleotides do not contain microhomologies sufficient for TMEJ (lanes 2 and 6). DCM10 was extended with Pol θ QM1 for 3 min in the presence of dGTP. Pol θ ΔCEN can use the newly extended DCM10 to pair with DCM19 to effectuate TMEJ (lanes 4 and 8). Conditions: stem–loop extension: 8 nM DCM10, 100 nM Pol θ QM1, and 50 μM dGTP. TMEJ: 8 nM SL-extended DCM10, 8 nM DCM19, 400 nM Pol θ ΔCEN, and 100 μM dNTPs. *C*, model describing how Pol θ can use stem–loop extension to create a microhomology to allow TMEJ in some situations. RPA binding to ssDNA to block stem–loop extension and TMEJ. The HLD (*cyan*) of Pol θ ΔCEN can use ATP or dATP to remodel the placement of RPA, restoring TMEJ and limited stem–loop extension activity. When the 3′-end cannot be used for end joining, the polymerase (*pink*) folds the 3′-end inside the active site and finds a preferred two-terminal base pairing upstream of the same strand to initiate stem–loop extension and synthesize a short sequence of DNA. Pol θ then releases the hairpin and uses the newly created 3′-end to search the other oligo for a MH site it can use as a primer to perform TMEJ. Repair products will then contain an inverted repeat, representing the newly created 3′-end. dNTP, deoxynucleotide; RPA, replication protein A; TMEJ, theta-mediated end joining.

SL extension has been inferred previously from analysis of TMEJ products *in vivo*. It was proposed that it acts to diversify the 3′-ends as an alternative to using an internal microhomology (37). A significant percentage of inverted repeats are found in the products of TMEJ in mammalian cells (38). We tested if the Pol θ QM1 extended products of DCM10, DCM1, and DCM9 oligonucleotides, respectively, could pair with designed terminally paired oligonucleotides to perform TMEJ. In all cases, the fully extended products of the Pol θ SL extension reactions formed stable hairpins that were resistant to denaturation in solution. This makes the newly created 3′-ends inaccessible for pairing with a second oligonucleotide. To circumvent this limitation, we took advantage of our finding that Pol θ QM1 can synthesize a new 3′-end composed of multiple guanines (Figs. 3*C* and 4*D*). The guanine-tailed oligonucleotides will not form a stable tight hairpin and will then be accessible to pairing with an oligonucleotide having five cytosines in its 3′-end (DCM19) for Pol θ ΔCEN to carry out TMEJ (Fig. 7*A*). We preincubated the enzyme with the DNA in low ionic strength solution to prevent the formation of G-quartet secondary structures. As expected, DCM10 is unable to pair efficiently with DCM19 and no TMEJ products are formed (Fig. 7*B*, lanes 2 and 6). However, after the G-tail was added to DCM10 through SL extension, Pol θ ΔCEN could join it with DCM19 to include the inserted sequence in the TMEJ products (Fig. 7*B*, lanes 4 and 8). A model depicting this mechanism is shown in Figure 7*C*. The 3′-ends of ssDNA can be available for Pol θ to use for a microhomology search as RPA binds with a 5′ to 3′ polarity. SL pairing will be limited to nearby pairing sites, sometimes creating a new microhomology. Pol θ can displace RPA from DNA using the HLD, to allow completion of TMEJ synthesis and repair.

## Discussion

DNA double-strand breaks are damaging events that can be repaired by several pathways, including homologous recombination, nonhomologous end joining, and TMEJ. While Pol θ usually causes deletions of a few base pairs during repair, its action prevents more catastrophic events such as large-scale deletions (1, 39). DNA sequence analysis of Pol θ–dependent TMEJ double-strand break repair events shows that inverted repeat sequences are often present at the repair junctions. Pol θ–mediated SL extension can, in principle, account for this finding, but the mechanism and rules governing this reaction have not been fully described. Here, we focused on the extension of ssDNA by purified human Pol θ *via* pairing of short internal microhomologies.

### Pol θ can extend ssDNA or ssRNA SLs

Pol θ is capable of SL extension of either DNA or RNA with dNTPs, whereas the related A-family DNA polymerase *E. coli* pol I Klenow fragment (Kf) cannot conduct this reaction. Pol θ is unable to extend RNA or DNA using ribonucleotides. Kf is similarly unable to incorporate ribonucleotides because Glu-710 and the aromatic side chain of Phe-762 act as a steric gate restricting the access of the incoming NTP (40). We confirmed that a variant in the analogous glutamate steric gate in Pol θ, Glu-2335 (16) allows the incorporation of ribonucleotides, although not as efficiently as dNTPs. Pol θ can also extend a DNA primer using an RNA template (20), a feature that is shared by other A-family DNA polymerases such as *E. coli* pol I Kf (19, 41). In fact, we find that Pol θ uses an RNA template much less efficiently than does *E. coli* pol I Kf exo^-^. It has been shown that steric-gate mutated Pol θ can give rise to essentially randomized sequence after several rounds of synthesis in the presence of Mn^2+^ (16), conditions known to greatly reduce the fidelity of DNA polymerases. It was first proposed that such synthesis is the result of a terminal transferase activity of Pol θ (16, 28), but it is now evident that such synthesis takes place in a templated fashion with a significant error-prone component in the presence of Mn^2+^ (13, 14). Although Pol θ forms SLs on RNA substrates less efficiently than on DNA, the reaction does proceed when dNTPs are available. RNA molecules including mRNAs, siRNAs, and long noncoding RNAs are abundant in cell nuclei, and such Pol θ–mediated reactions could conceivably occur in cancer cells where Pol θ is highly expressed.

### Pol θ SL synthesis has a rapid high-fidelity phase and a slower low-fidelity phase

An important finding of the present studies is that SL extension by Pol θ occurs in two phases. The first phase occurs quickly and follows base-pairing rules. Given more time, Pol θ mediates further slower reactions that expand the diversity of products. Pol θ is able to synthesize these products due to its tolerance for primer-template slippage (8) and mismatch incorporation (9). Our work has focused on normal conditions with the physiological Mg^2+^ divalent cation but, even with Mg^2+^, there is significant slippage when dNTP choice is restricted. At least two terminal base pairs are required for efficient SL extension. If the nucleotide pool is restricted to a single dNTP, it is notable that Pol θ can use a stable terminal pairing site to repeatedly extend using the available nucleotide. Such slippage events still depend on the terminal base pairing of an internal SL, as slippage products arise only with the nucleotide complementary to the template following the predicted pairing sites (Fig. 3*C*). The implications for Pol θ′s function *in vivo* may be profound. Given that previous studies of purified Pol θ were largely conducted with longer time points (5–30 min), Pol θ is likely to operate more quickly *in vivo* and with higher fidelity than sometimes appreciated.

### Efficient SL synthesis by Pol θ

Here, we show that Pol θ has the unusual ability to manipulate a short oligonucleotide so that the 3′ terminus can initiate synthesis in a unimolecular reaction. The pairing inside the enzyme would otherwise be thermally unstable in solution. There must be specific features of Pol θ that coordinate quick and efficient SL formation and extension, because the closely related Kf and human Pol ν enzymes cannot perform SL synthesis. The crystal structure of Pol θ in complex with a primer-template shows specific contacts with the incoming nucleotide and the five backbone phosphates nearest to the 3′-end of the primer strand (1). Correspondingly, it is notable that all the SL structures preferred by Pol θ have a primer strand of 5 to 6 nt before the loop begins. Three of the residues (K2181, R2202, and R2254) that contact the primer are unique to Pol θ and not present in other homologous A-family DNA polymerases. We found that in the 3A triple variant K2181A, R2202A, R2254A lacking these three “primer-grasp” contacts, SL extension was 6-fold slower, indicating that these three residues help coordinate this reaction. To date, there is one electron microscopy structure of a complex of a fish (*Lates calcacifer*) Pol θ bound to a DNA SL (42). The three analogous basic residues (K2395, R2343, and K2322) in fish Pol θ contact the DNA near the 3′-end, at positions n-1 and n-2, relative to the base pair in the active site. This arrangement is reminiscent of the contacts observed with recessed primer/templates where the protein contacts are localized near the 3′-end of the primer (25, 43, 44). While the stem is clearly defined in the structure, the loop itself is not (42). Further structural work is therefore warranted to reveal how Pol θ manipulates and extends SLs.

We found that Pol η can perform SL extension on some oligonucleotides, although with preferences different from Pol θ. Pol η has a spacious active site that can accommodate DNA adducts and non-Watson–Crick base pairs with little discrimination (45). This allows Pol η to incorporate base adducts during translesion DNA synthesis. It is possible that the SL activity of Pol η might be used in cells and account for some insertions observed during DSB repair. Pol η can also synthesize on an RNA template (46, 47, 48) and is implicated in DSB repair using an RNA scaffold (11). Further, Pol η can replicate through G-quadruplex DNA structures, often producing insertions of a single base, or more complex insertion events (49). Some of these might be accounted for by the ability of Pol η to manipulate a primer end into a SL. Pol λ can also perform SL extension on some oligonucleotides (Figs. 4*C* and S4*B*). Pol λ is involved in nonhomologous end joining in cells (50) and it is conceivable that SL formation may help to diversify base pairing options for strand break repair.

### Reconstitution of a TMEJ event containing a templated insertion

DNA sequencing experiments from multiple organisms show that TMEJ repair events frequently include short insertions of DNA sequence. Evidence of Pol θ–derived templated insertions (TINs) has been found in plants (51), invertebrates including *Caenorhabditis elegans* (39) and vertebrates (52). In *Drosophila*, TINs are the most frequent products of TMEJ (53). TINs contain direct or inverted repeat copies of adjacent (or sometimes more distant) sequences. One or more regions of local template are copied to create a new terminus before repair is completed (12, 37, 54).

An inverted TIN likely arises from a short DNA synthesis event when a 3′-end initiates copying from DNA behind it on the same strand. The signature of Pol θ–mediated inverted TINs induced by Cas9-DSBs has been characterized in *Drosophila* (55, 56), where pairing sites of 1 to 3 bp separated by a 6-nt loop were most favorable for initiation of inverted TINs (55). Inverted TINs of 3 to 30 bp, with sequence identical to flanking DNA, are also frequently observed in mammalian cells (38). These inverted TINs are more prevalent when microhomology near the ends of the initial break is poor. TINs may contribute to break repair by potentially introducing a new microhomology when a preexisting choice is not available (55, 56). In analyses of genomic changes in cancers, TINs are a more specific biomarker of TMEJ activity than microhomology-associated deletions (38, 57, 58).

Production of TINs during TMEJ is likely tightly regulated in the cell to prevent uncontrolled extension of single-stranded 3′-ends. In mammalian cells, inverted repeat TINs have been reported in less than 7% of repair sites that have no microhomologies of ≥3 bp within 15 nt of the initial break site (38). The resected 3′-ends at the DSBs will not remain exposed for long. In cells, RPA will quickly coat the ssDNA ends to protect them from extended resection. We hypothesized that RPA could also act to cover the SL pairing sites and block SL extension. Using human recombinant proteins, RPA blocks SL extension when present in high concentrations relative to DNA. ATP- or dATP-dependent action of the HLD of Pol θ can reverse some of this inhibitory effect, displacing RPA from DNA. SL-mediated insertions may be more significant in homologous recombination-deficient cancers, which are more dependent on Pol θ. In tumors that express Pol θ at higher levels and rely on TMEJ, an increased incidence of mutagenic TINs might confer an advantage to some cancer cells.

An unsolved problem is how extended hairpin products are unraveled and sometimes incorporated into repair products in the cell. We show that this can occur because some hairpin products are formed by repeated slippage events. We took advantage of this capability of Pol θ by generating a SL extension product to create a new MH *in vitro* that could then be used to reconstitute insertion-mediated TMEJ (Fig. 7*B*). This finding shows that purified Pol θ can conduct SL synthesis that can be incorporated into TMEJ products. It is also possible that another, yet unidentified, component may help Pol θ release the hairpin product to allow for TMEJ. Future work may identify other factors that participate in SL extension.

## Experimental procedures

### Enzymes and oligonucleotides

Human DNA Pol θ QM1 (1792–2590), E2335G variant and 3A variant (K2181A, R2202A, R2254A) (Doublié Lab). Human DNA Pol θ full-length (Wood Lab), human DNA Pol θ ΔCEN (Wood lab). DNA Pol ν (Wood Lab). Human DNA Pol η (Enzymax Cat. No. 19). Human DNA Pol γ (Enzymax Cat. No. 85). Human DNA Pol κ (Enzymax Cat. No. 27). Human DNA Pol ι (Enzymax Cat. No. 20) Human DNA Pol β (Enzymax Cat. No. 22). Human DNA Pol μ (Enzymax Cat. No. 21). RB69 exo (−) (Doublié Lab). Human DNA Pol β (Doublié Lab). Human RPA (Doublié Lab). KF (3’→5′ exo^-^) (New England Biolabs Cat. No. M0212L). Pol λ (catalytic domain V235-W575) was a gift from Lars Pedersen, National Institute of Environmental Health Sciences (50). Oligonucleotides used in figures are synthesized from integrated DNA technologies and listed in the Table S1.

### Purification of proteins

The DNA polymerase domain of Pol θ (QM1, residues 1792–2590) was purified (Fig. S1*C*) after expression in *E. coli* as described (25, 59). Constructs for expression of variants E2335G and 3A (K2181A, R2202A, R2254A) were synthesized by GenScript and purified in the same manner. Human full-length Pol θ was purified after expression in human Expi293 cells (18, 60). The Pol θ ΔCEN construct replaced residues 911 to 1791 with a flexible linker of sequence GSAGSAAGSGEF and was purified in a similar manner (Fig. S1*C*).

### Reagents

dNTPs (dATP, dCTP, dTTP, dGTP) (Thermo Fisher Scientific Cat. No. 10297018). Ribonucleotides (NTPs) (ATP, CTP, TTP, GTP) (Thermo Fisher Scientific Cat. No. R0481). Dideoxynucleoside triphosphate set (ddATP, ddCTP, ddTTP, ddGTP) (Millipore Sigma Cat. No. 03732738001). Magnesium chloride (MgCl_2_) (Thermo Fisher Scientific Cat. No. 68475). Potassium phosphate monobasic (Sigma-Aldrich Cat. No. P5655), potassium phosphate dibasic (Cat. No. P8281). Bovine serum albumin (Cell Signaling Cat No. 9998S). DL DTT (Sigma-Aldrich Cat. No. D0632). ZipTip with 0.6 μl C_18_ resin (Millipore Sigma Cat. No. ZTC18S096). Triethylamine acetate 2.0 M (Thermo Fisher Scientific Cat No. 400771). Acetonitrile (Thermo Fisher Scientific Cat. No. 047138.K2). UltraPure DNase/RNase-free distilled water (Thermo Fisher Scientific Cat. No. 10977015). EvaEZ fluorometric polymerase activity assay kit (29051, Biotium).

### Buffers

DNA synthesis buffer (EZX Buffer): 25 mM potassium phosphate (pH 7), 5 mM MgCl_2_, 5 mM DTT, 0.1 mg/ml bovine serum albumin. 2× denaturing stop buffer: 20 mM EDTA in 95% formamide containing bromophenol blue. 6X TMEJ stop buffer: 300 mM Tris–HCl pH 7.5, 3 mg/ml proteinase K, 120 mM EDTA, and 1.2% SDS. TMEJ nondenaturing loading buffer: 40% sucrose with 0.01% bromophenol blue.

### Polymerase activity assay

Polymerase Unit/ml of Pol θ full-length protein, Pol θ ΔCEN, and Pol θ QM1 were calculated using EvaEZ fluorometric polymerase activity assay kit (29051, Biotium). In brief, a serial dilution curve of Kf exo^-^ (M0212, NEB) was prepared and equally mixed well with 2× EvaEZ polymerase activity mix on ice. The quantitative PCR reaction was started after placing the mixture at 37 °C. The fluorescence of each dilution was monitored every 30 s. The polymerase activity of each Kf exo^-^ dilution was assessed by calculating the slope of the initial linear portion of the curve. A standard curve was generated by plotting the initial rate of fluorescence increase for each concentration against the serial activity unit. We then calculated the polymerase activity of each enzyme as follows: Pol θ full-length protein 1.35 U/ml (100 nM), Pol θ ΔCEN 8 U/ml (100 nM), and Pol θ QM1 5.38 U/ml (100 nM).

### Single strand and primer extension assays

ssDNA oligonucleotides, RNA, or primed templated DNA was incubated with human Pol θ in 1× EZX buffer containing deoxy-ribonucleotides, ribonucleotides, or dideoxynucleotides. Reaction mixtures were incubated at 37 °C. Times and DNA/enzyme concentrations are given in the Figure legends. Reactions were terminated by addition of denaturing stop buffer and heated to 95 °C before loading for gel electrophoresis. Samples were run on a gel containing 20% 19:1 bis-acrylamide and 7 M urea at 450 V/cm using Bio-Rad PROTEAN XL gel running system. Gels were scanned using a Typhoon Biomolecular Imager (Amersham) with detection set for Cy5 or 6-FAM fluorescence.

### TMEJ assay

Oligonucleotide pairs were incubated together with DNA Pol θ QM1 or Pol θ ΔCEN at 37 °C for 10 min in 1× EZX buffer. dNTPs (100 M) were then added to start the polymerase reactions for several timepoints. The reactions were stopped with TMEJ stop buffer and incubated at 37 °C for 30 min. 1× loading buffer was added and samples were further incubated at 37 °C for 10 min. Samples were run on 10%, 15%, or 20% 19:1 bis-acrylamide native gels depending on the oligo length. Gels were scanned using the Typhoon imager with detection set for Cy5 fluorescence, Cy3 fluorescence, or 6-FAM.

### TMEJ *versus* SL competition assay

25 nM Cy5 5′-labeled 27-nt DCM16-2 or DCM17 in 1x EZX buffer and 200 nM Pol θ ΔCEN were incubated at 37 °C for 3 min. Addition of ddATP or ddCTP in the dNTP mix will terminate the end-joining product to 31 nt while leaving the SL extension unchanged. Reactions were started by addition of 100 μM dNTPs and incubated at 37 °C for 15 min. Reactions were stopped by adding 1× denaturing stop buffer and boiled at 95 °C. Samples were separated on a gel containing 20% 19:1 bis-acrylamide and 7 M urea (450 V, 3.5 h). Gels were scanned using the Typhoon imager detecting Cy5 fluorescence.

### RPA inhibition assay

Oligonucleotides (6.4 nM of 50-mer ssDNA) were incubated with increasing concentrations of human RPA (8, 16, 32, 64, and 128 nM) at 37 °C for 15 min in the presence or absence of 1 mM ATP. Oligonucleotide 6.4 nM of Pol θ ΔCEN and Pol θ QM1 was added to the reaction in 1× EZX buffer containing 4 μM dNTPs. Reactions were further incubated at 37 °C for 10 min. Reactions were terminated by addition of denaturing stop buffer. Samples were run on gels containing 20% 19:1 bis-acrylamide and 7 M urea. In Figure 6*B*, 25 nM DCM12, 25 nM DCM13, and 1× EZX buffer were incubated at 37 °C with different concentrations of RPA (0, 0.1, 0.2, 0.4, and 0.8 μM). For reactions with ATP, the initiation mixture was 100 μM dNTPs and 5 mM ATP. For reactions without dATP, the initiation mixture was 100 μM dCTP, dGTP, and dTTP. After 15 min, 200 nM Pol θ ΔCEN is added to each tube and incubated for 3 min at 37 °C. The polymerization reaction was initiated with 100 μM of dNTPs and incubated for 30 min at 37 °C. The reaction was terminated with 1× TMEJ stop buffer and incubated for 30 min at 37 °C. 1× loading dye with sucrose was added, and samples were incubated at 37 °C for 10 min. The samples were loaded into a 10% polyacrylamide gel and ran at 200 V for 2 h. Gels were scanned using the Typhoon imager detecting Cy5 and Cy3 fluorescence.

### Pol θ creates microhomology to aid TMEJ

The first reaction was with 10 nM 5′-Cy5-DCM10 oligonucleotide (25 nt), 100 nM Pol θ QM1, and 25 μM dGTP in 1× EZX buffer. Reactions without Pol θ QM1 were also prepared. Reactions were incubated at 37 °C for 5 min. Reactions were stopped by denaturing QM1 after boiling samples at 95 °C for 5 min. DNA was then purified using ZipTipC18 0.6 μl resin tips. Purification will remove Pol θ QM1 enzyme and unincorporated dGTPs. The purified DNA was then transferred to a second tube and incubated at 37 °C for 10 min with 10 nM 5ʹ-FAM-DCM19 oligo and 40 nM Pol θ ΔCEN enzyme in nuclease free water. Subsequently a second reaction was initiated by addition of 100 μM dNTPs and the 1× EZX buffer and incubated at 37 °C for 30 min. The salt in the EZX buffer might help stabilize G-quartet secondary structures. Therefore, it was left out of the reaction until addition of the dNTPs. Reactions were stopped by addition of 6× TMEJ stop buffer and incubated at 37 °C for 30 min. 1× loading dye containing sucrose was added and reactions were further incubated at 37 °C for 10 min to complete the proteinase K reaction. Samples were then run on a 15% 19:1 bis-acrylamide gels native gels at 300 V for 2.5 h. Gels were scanned using the Typhoon imager with detection set for Cy5 fluorescence and 6-FAM fluorescence.

### Densitometry and statistical analysis

For statistical analysis of single strand extension of RNA oligonucleotides DCM2 and DCM10, reactions were run on the same day, combined, and plotted together for an experimental n = 2 in Figure 1*D*. For statistical analysis of Pol θ 3A variant and Pol θ QM1 reaction kinetics, reactions were run in triplicate on three different days with different enzyme aliquots for an experimental n = 3. The pixel intensity of each band present in each lane was tabulated. The original substrate band was identified and set as starting value for each reaction. All the other bands present above the original substrate band were considered extended products. In the primer/template reactions the band corresponding to the last templated base +1 was set as the full extension. The sum of the pixel intensity from all the bands in each lane was set to 100%. Percent SL extension and primer/template extension was calculated by subtracting the original substrate pixel intensity from the sum of all the extended products bands present in each lane and calculating the percentage based 100% pixel intensity. The percent extension values for each experiment were plotted using Prism (GraphPad, https://www.graphpad.com), version 8.0.4. Data was plotted as mean ± SD. The best fit curve was obtained with a nonlinear regression equation:

Percent extension = 100 × t^h^/(K_half_^h^ + t^h^), where t = time, K_half_ is the time at a half completion, and h is a Hill slope (empirical measure of the steepness of the curve). Significance was obtained by doing a paired *t* test on the initial rate of reaction from the individual experiments.

Densitometry analysis was performed for the experiments in Figure 5, *A*, *B*, and *D*, to assess the percent TMEJ and SL extension products formed in the various reaction conditions. Statistical analysis was not performed as these are individual single experiments. The pixel intensity of each band present in each lane was tabulated. The original substrate band was identified and set as starting value for each reaction. All the other bands present above the original substrate band were considered either TMEJ products or SL extension products depending on the color of the band. Only the bands that present as dark blue in the merged image were considered TMEJ products bands. All the other bands that migrate above the original substrate bands that present as either cyan or purple bands were considered SL extension products. The sum of the pixel intensity from all the bands in each lane was set to 100%. Percent SL extension and TMEJ was calculated comparing the pixel intensity for each product band and comparing that to the total pixel intensity of the all the bands in the lane previously set to 100%. The percentage of remaining original substrate was also calculated the same way. The percent of TMEJ extension, SL extension, and remaining substrate for each condition was plotted using PRISM Graph Pad Version 8.0.4. The percentage calculated for each condition was included in the bar graph for clarity.

## Data availability

All data from this study are included in the manuscript and Supporting information.

## Supporting information

This article contains supporting information.

## Conflict of interest

R. D. W. owns stock in Repare Therapeutics Inc. The other authors declare that they have no conflicts of interest with the contents of this article.
