# Supplementary material for: Dynamic stem–loop extension by Pol θ and templated insertion during DNA repair
Source: J Biol Chem. 2024 Jun 12;300(7):107461. doi: 10.1016/j.jbc.2024.107461 (PMC11292364; doi:10.1016/j.jbc.2024.107461)
Supplement: Supporting figures [file mmc1.docx]

**Supporting Information**

**Dynamic stem–loop extension by Pol θ and templated insertion during DNA repair**

Denisse Carvajal-Maldonado^1^, Yuzhen Li^1^, Mark Returan^1^, April M. Averill^2^,

Sylvie Doublié^2^ and Richard D. Wood^1,5^

^1^Department of Epigenetics and Molecular Carcinogenesis, The University of Texas MD Anderson Center, Houston, Texas, USA; email: [rwood@mdanderson.org](mailto:rwood@mdanderson.org)

^2^Department of Microbiology and Molecular Genetics, University of Vermont, Burlington, Vermont, USA; email: [sdoublie@uvm.edu](mailto:sdoublie@uvm.edu)

^5^Corresponding author

Running title:  *Extension of DNA and RNA oligonucleotides by Pol θ*

Keywords:

*DNA repair, DNA synthesis, protein‐DNA interaction, RNA synthesis, DNA polymerase*

**
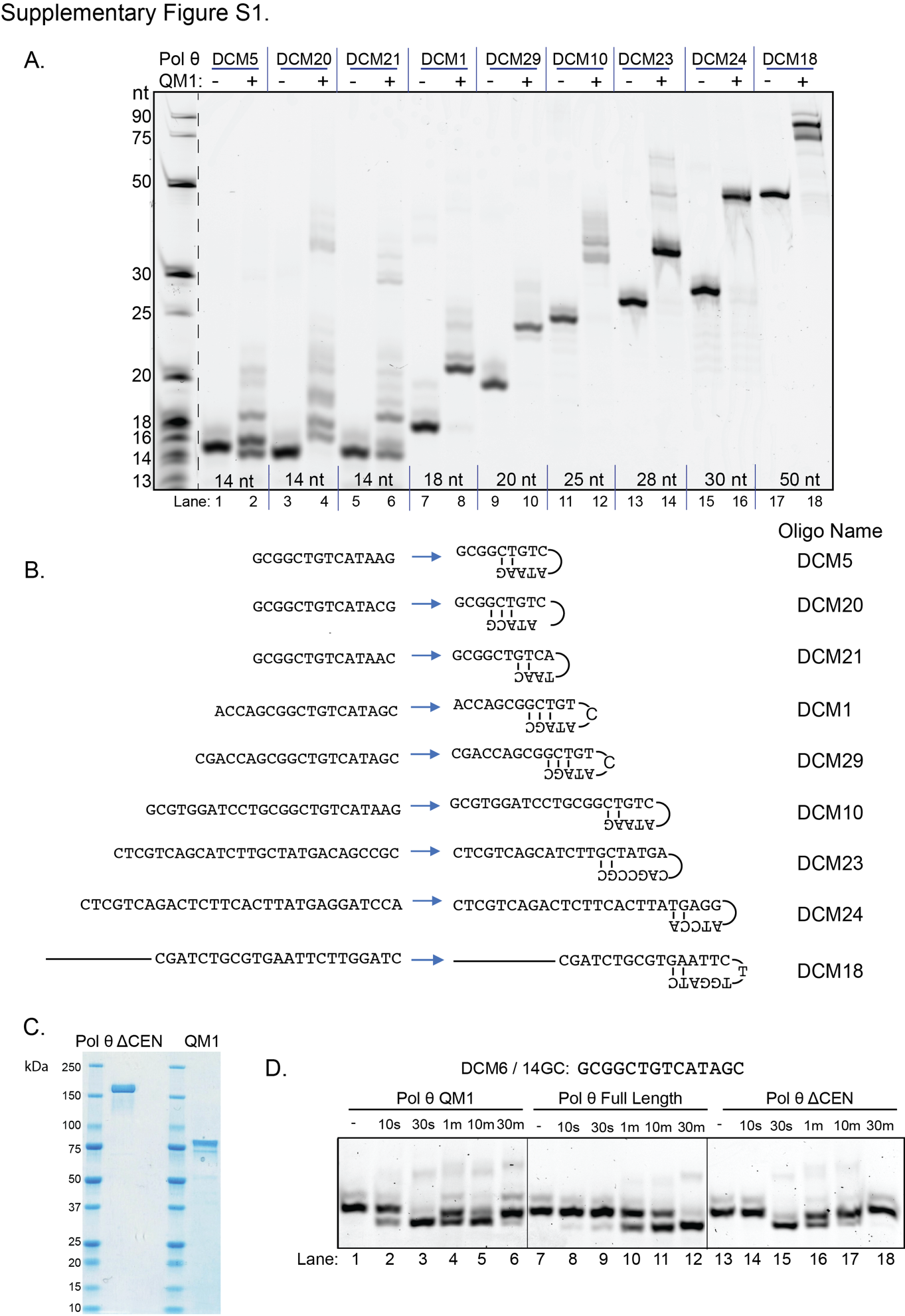
**

**Supplementary Figure S1** A) Stem-loop extension of Cy5-labeled ssDNA oligonucleotides of various sizes using Pol θ QM1 (+) or no enzyme (-). The dotted line following the marker lane (nt) indicates where four lanes were spliced from the gel before the data in the following lanes. B) Potential stem-loop pairing sites of oligonucleotide in (A) that can be used to extend by Pol θ QM1. Conditions: 25 nM DNA, 125 nM enzyme and 100 µM dNTPs. C) Coomassie blue-stained polyacrylamide gel showing the purified human Pol θ QM1 (90 kDa) and Pol θ ΔCEN proteins (193 kDa) used here. D) Extension of DCM6 using Pol θ QM1, Pol θ full-length and Pol θ ΔCEN proteins. After 30 s to 1 min of incubation, all three enzymes form faster migrating products on a 20% polyacrylamide gel containing 7 M urea. Conditions: 25 nM DNA, 125 nM Pol θ and 100 mM dNTPs.


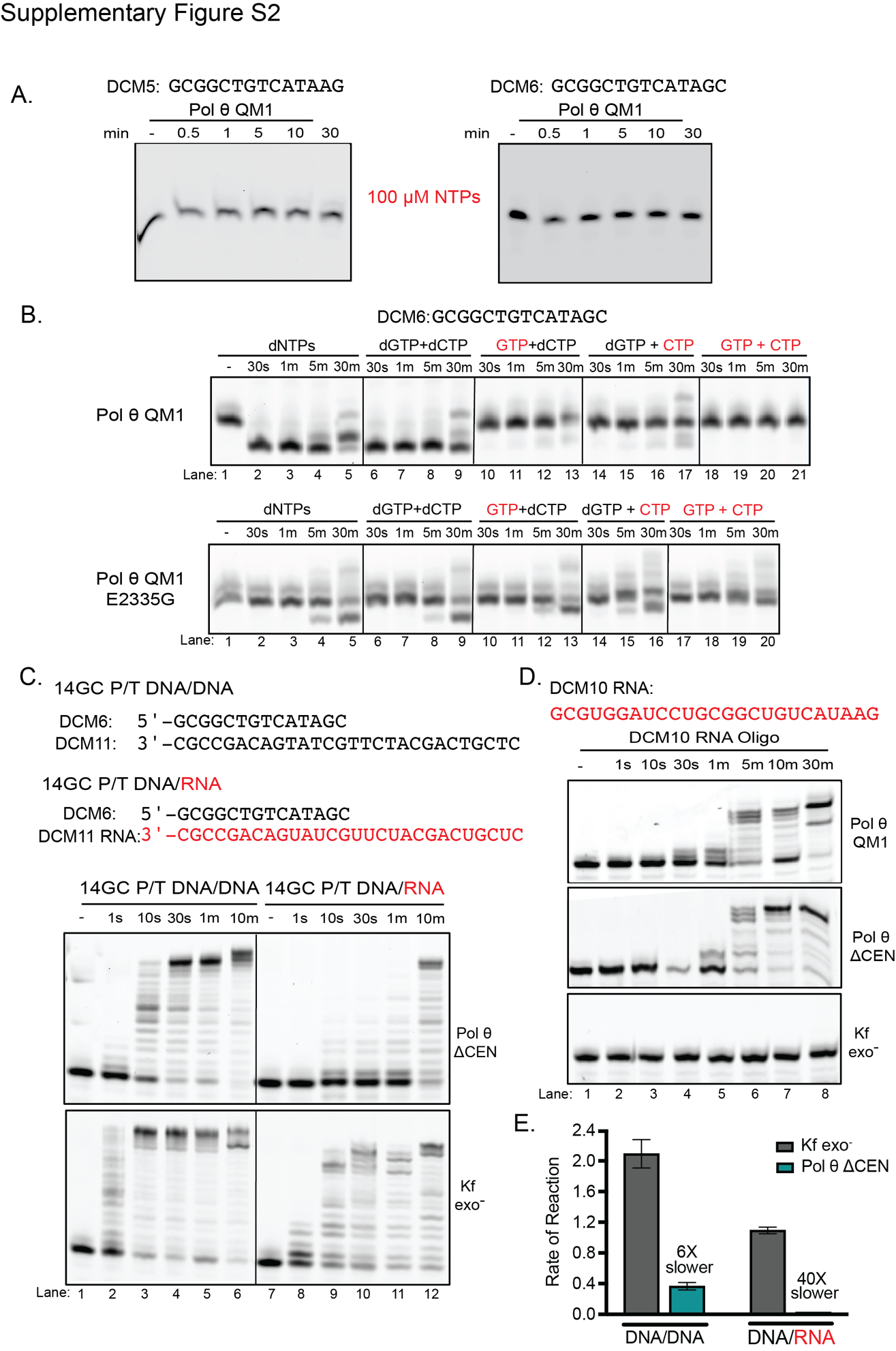


**Supplementary Figure S2.** A) NTPs (100 μM) do not support extension of ssDNA oligonucleo­tides (DCM5 and DCM6) by Pol θ QM1. B) Stem-loop extension of DCM6 oligonucleotides using Pol θ QM1 (top) and Pol θ QM1 E2335G variant (bottom) with either dNTPs, dGTP+dCTP, GTP + dCTP, dGTP + CTP and GTP + CTP nucleotides. C) Extension of DNA/DNA (DCM6/DCM11, left) and DNA/RNA (DCM6/DCM11 RNA, right) primer/template substrates using Pol θ ΔCEN (top) and Kf exo^-^ (bottom). D) Stem-loop extension of DCM10 RNA using Pol θ QM1 (top), Pol θ ΔCEN (middle) and Kf exo^-^ (bottom). E) Quanti­fication of primer-template reaction rates for each enzyme based on the K_1/2_ of the plots in Figure 1F. Conditions for all reactions: 25 nM DNA or 25 nM RNA, 125 nM enzyme, 100 μM dNTPs.

**
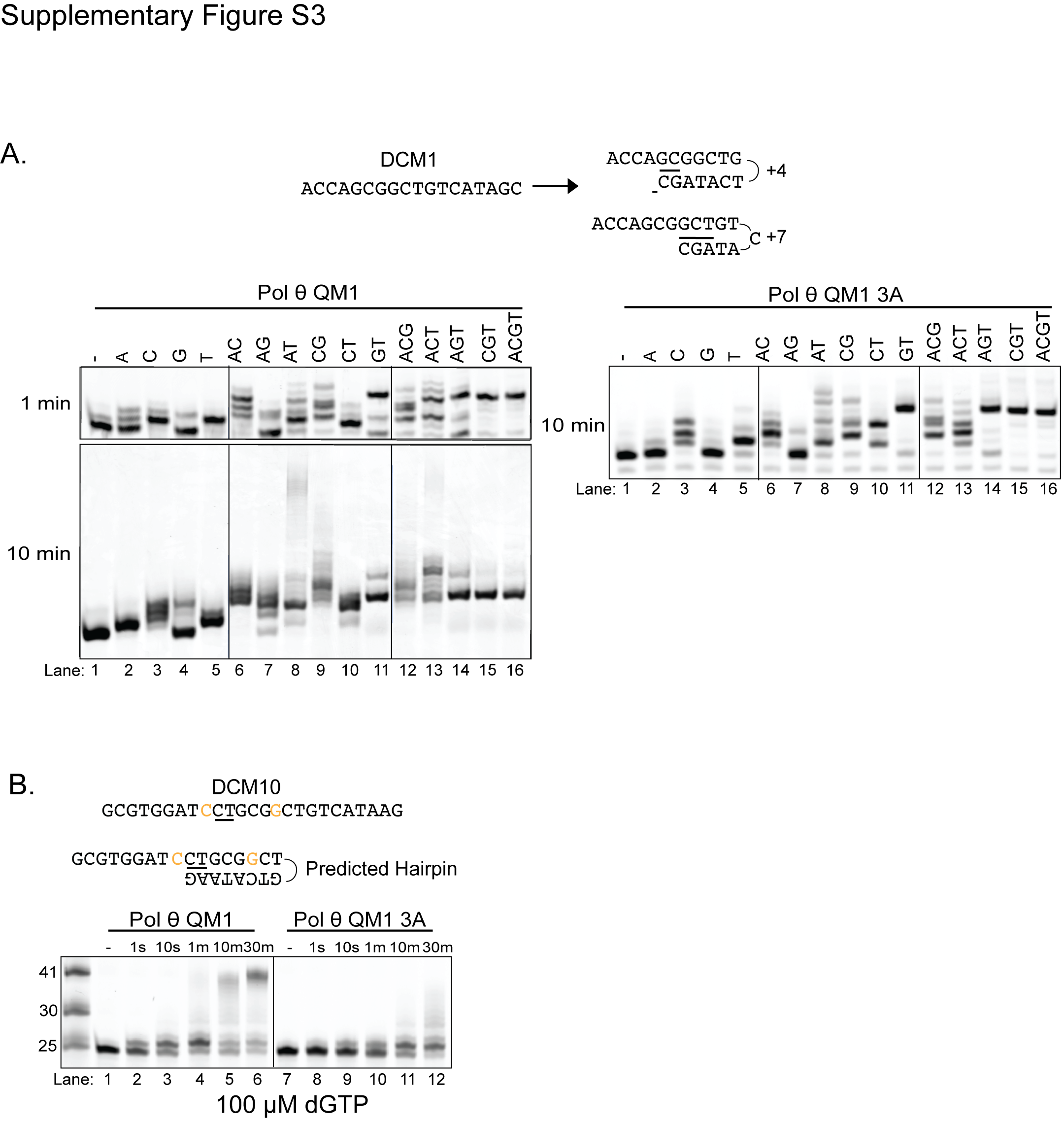
**

**Supplementary Figure S3**. A) Pol θ QM1 stem-loop extension of DCM1 using different combinations of dNTPs in 1-min and 10-min reactions. Schematic of hairpins used by Pol θ QM1 in the short (left top) and the long reactions (left bottom). The 10-min reaction with Pol θ QM1 is the same gel as shown in Figure 4C. Pol θ QM1 3A variant stem-loop extension of DCM1 in 10-min reactions (right). B) Comparing Pol θ QM1 (left) and Pol θ QM1 3A variant (right) stem-loop extension slippage activity using DCM10.

**
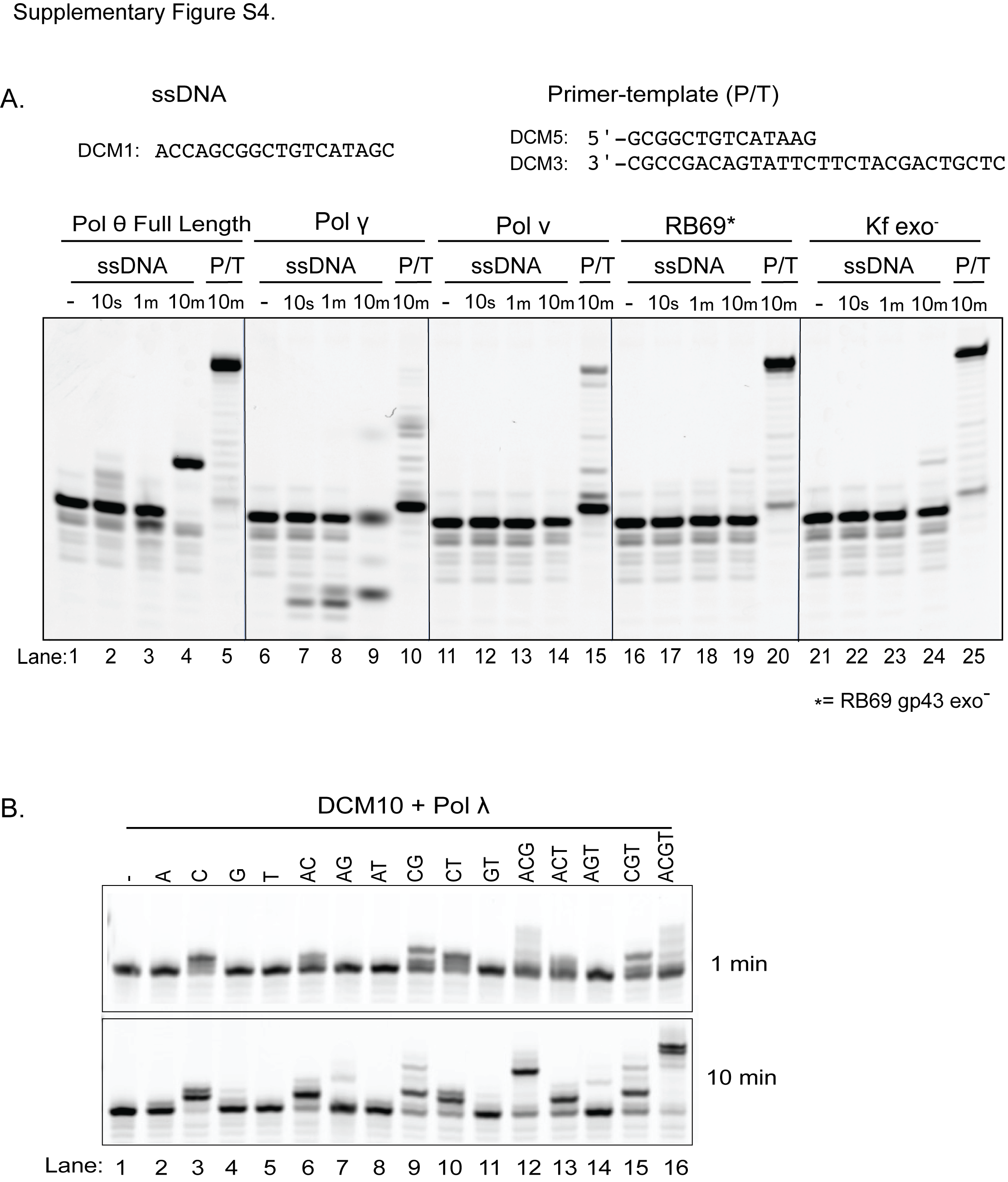
**

**Supplementary Figure S4.** Stem-loop extension of DCM1 and DCM5/DCM3 primer-template extension using A-family DNA polymerases (Pol θ Full Length, Pol γ, Pol ν and Kf exo^-^ and B-Family polymerase RB69 exo^-^. ssDNA was incubated with the various enzymes for different time points (10 s, 1 min and 10 min). The primer-template substrate was incubated with the enzymes for 10 min as a positive control for polymerase activity. Conditions: 25 nM DNA, 125 nM enzyme and 100 µM dNTPs. B) Stem-loop extension of DCM10 using Pol λ with different combinations of dNTPs in 1-min and 10-min reactions.
