# Supplementary material for: Dynamic stem–loop extension by Pol θ and templated insertion during DNA repair
Source: J Biol Chem. 2024 Jun 12;300(7):107461. doi: 10.1016/j.jbc.2024.107461 (PMC11292364; doi:10.1016/j.jbc.2024.107461)
Supplement: Table S1 [file mmc2.docx]

Supplementary Table S1 Oligonucleotides sequences

| **Stock Oligo Name** | **Oligo Names** | **5'-end** | **5'-Sequence-3'** | **3' end** | **Length** | **DNA/RNA** | **Figure Used** |
| --- | --- | --- | --- | --- | --- | --- | --- |
| 14e + 4 (ACCA-14e) (166-A1) (18MER) | DCM1 | Cy5 | ACCAGCGGCTGTCATAGC | 3'-OH | 18 | DNA | 1B, 3D, 4A-C, S1A-B, S3A, S4A |
| ACCA-14e RNA | DCM2 | Cy5 | ACCAGCGGCUGUCAUAGC | 3'-OH | 18 | RNA | 1C |
| 28-14a | DCM3 | None | CTCGTCAGCATCTTCTTATGACAGCCGC | 3'-OH | 28 | DNA | 1E-F, 4A-B, S4A |
| Cy5-28-14a | DCM23 | Cy5 | CTCGTCAGCATCTTCTTATGACAGCCGC | 3'-OH | 28 | DNA | S1A-B |
| 14a-28 RNA | DCM3 RNA | None | CUCGUCAGCAUCUUCUUAUGACAGCCGC | 3'-OH | 28 | RNA | 1E-F |
| Cy5-14a | DCM5 | Cy5 | GCGGCTGTCATAAG | 3'-OH | 14 | DNA | 1E-F, 4A-B, S1A-B, S2A, S4A |
| ss14e-Cy5 | DCM6 | Cy5 | GCGGCTGTCATAGC | 3'-OH | 14 | DNA | 2A-C, 3D, S1D, S2A-C |
| DCM6-FAM-PHOS | DCM6-P | 6-FAM | GCGGCTGTCATAGC | 3'-PO4 | 14 | DNA | 2A-C |
| Fw20nt JC/DR (3 JC/DR) | DCM9 | Cy5 | AGAACCAAGATGCAGTTTTT | 3'-OH | 20 | DNA | 3A-B |
| Cy5-14a-NGSr/14a+BamH1 | DCM10 | Cy5 | GCGTGGATCCTGCGGCTGTCATAAG | 3'-OH | 25 | DNA | 3C, 4D, 7A-B, S1A-B, S3B, S4B |
| 14a + BamH1 RNA | DCM10 RNA | Cy5 | GCGUGGAUCCUGCGGCUGUCAUAAG | 3'-OH | 25 | RNA | S2D |
| 14e-28 | DCM11 | None | CTCGTCAGCATCTTGCTATGACAGCCGC | 3'-OH | 28 | DNA | 3D, S2C |
| 14e-28 RNA | DCM11 RNA | None | CUCGUCAGCAUCUUGCUAUGACAGCCGC | 3'-OH | 28 | RNA | S2C |
| Cy5-90-6MH-1 | DCM12 | Cy5 | TGACTATACAGCTAAGGGATCCTCTCACCGAGCGTATCTGCTGGGTTGTGGATGAATTACATATGCTGGGAGAACCAAGATTGGGCAGGG | 3'-OH | 90 | DNA | 5A, 6B |
| Cy3-90-6MH-7 | DCM13 | Cy3 | AGTCTGAGCTCGGTGTGAGAGTGAAGATCCTCACCTTCGGAGTACTCCTTCTTTTGACCATTGATACGATACTTCTCAACCGAGCCCTGC | 3'-OH | 90 | DNA | 5A, 6B |
| CF36CCColigo | DCM14 | Cy5 | TGGCGACGGCAGCGAGGCCCCCCCCCCCCCGCACAG | 3'-OH | 36 | DNA | 5B |
| 188-1 primer CF | DCM14Temp | None | GCCTCGCTGCCGTCGCCA | 3'-OH | 18 | DNA | 5B |
| 188-3 Primer CF | DCM15Temp | None | TGGCGACGGCAGCGAGGC | 3'-OH | 18 | DNA | 5B |
| CF-36TTT oligo | DCM15 | 6-FAM | GCCTCGCTGCCGTCGCCATTTTTTTTTTTTCTGTGC | 3'-OH | 36 | DNA | 5B |
| Oligo TII27-mer | DCM16-2 | Cy5 | TGCAGCAGACACGATACTCGCTACGTA | 3'-OH | 27 | DNA | 5C-E |
| Oligo GII27-mer | DCM17 | Cy5 | GTACTACTCACATCGCAGATAGCATGC | 3'-OH | 27 | DNA | 2D-F, 5D |
| GI41-mer | 41 nt Marker | Cy5 | GTACTACTCACATCGCAGCTAGCATGCGATGTGAGTAGTAC | 3'-OH | 41 | DNA | 2D-F |
| Marker-GII27-30 | 30bp Marker | Cy5 | GTACTACTCACATCGCAGATAGCATGCGAT | 3'-OH | 30 | DNA | 2D-F |
| Marker-GII27-30R&C |  | None | ATCGCATGCTATCTGCGATGTGAGTAGTAC | 3'-OH | 30 | DNA | 2D-F |
| FAM-DCM6COMP-PHOS TRUE | DCM22 | 6-FAM | GCTATGACAGCCGC | 3'-OH | 14 | DNA | 2C |
| G4-27 | DCM17-4 | 6-FAM | GTACTACTCCGATCGCAGATAGCATGC | 3'-OH | 27 | DNA | 2D-F |
| Cy5-Marker 50bp | DCM18 | Cy5 | ACACTCTTTCCCTACACGACGCTCTTCCGATCTGCGTGAATTCTTGGATC | 3'-OH | 50 | DNA | 6A, S1A-B |
| 166-D5 | DCM19 | 6-FAM | CTTATCGACTATTGTTCATCTGTGAGTTACCTTGCAAATATATATCCCCC | 3'-OH | 50 | DNA | 7A-B |
| ss14CG | DCM20 | Cy5 | GCGGCTGTCATACG | 3'-OH | 14 | DNA | S1A-B |
| ss14AC | DCM21 | Cy5 | GCGGCTGTCATAAC | 3'-OH | 14 | DNA | S1A-B |
| Cy5-14ag-30 | DCM24 | Cy5 | CTCGTCAGACTCTTCACTTATGAGGATCCA | 3'-OH | 30 | DNA | S1A-B |
| 14e-20MER | DCM29 | Cy5 | CGACCAGCGGCTGTCATAGC | 3'-OH | 20 | DNA | S1A-B |
